# Physical activity in gestational trimesters and perinatal outcomes in SUS puerperal women

**DOI:** 10.11606/s1518-8787.2021055003067

**Published:** 2021-10-13

**Authors:** Tauana Prestes Schmidt, Talita Tuon, Katia J. Pudla Wagner, Antonio Fernando Boing, Ana Lúcia Danielewicz

**Affiliations:** I Universidade Federal de Santa Catarina Programa de Pós-Graduação em Ciências da Reabilitação AraranguáSC Brasil Universidade Federal de Santa Catarina. Programa de Pós-Graduação em Ciências da Reabilitação. Araranguá, SC, Brasil; II Universidade Federal de Santa Catarina CuritibanosSC Brasil Universidade Federal de Santa Catarina. Coordenadoria Especial de Biociências e Saúde Única. Curitibanos, SC, Brasil; III Universidade Federal de Santa Catarina Departamento de Saúde Pública FlorianópolisSC Brasil Universidade Federal de Santa Catarina. Departamento de Saúde Pública. Florianópolis, SC, Brasil; IV Universidade Federal de Santa Catarina Departamento de Ciências da Saúde AraranguáSC Brasil Universidade Federal de Santa Catarina Departamento de Ciências da Saúde. Araranguá, SC, Brasil

**Keywords:** Pregnant Women, Exercise, Natural Childbirth, Cesarean Section, Premature Birth, Cross-Sectional Studies

## Abstract

**OBJECTIVE:**

Test the association between the practice of physical activity (PA) according to the gestational trimesters and the occurrence of cesarean delivery, prematurity, and low birth weight in puerperal women assisted in the Unified Health System of Santa Catarina, Brazil.

**METHODS:**

A cross-sectional study was conducted with a probabilistic sample of puerperal women who gave birth in public maternity hospitals in Santa Catarina from January to August 2019. The cesarean delivery outcome was self-reported, and information on premature birth (< 37 gestational weeks) and low birth weight (< 2,500 grams) were obtained from medical records. The practice of PA during pregnancy and according to each trimester was self-reported. Multivariate Logistic Regression analyses and interviews with 3,580 puerperal women were carried out.

**RESULTS:**

PA practice during any period of pregnancy was reported by 20.6% of the sample, with a gradual reduction in prevalence according to the gestational trimester (16.2%, 15.4%, and 12.8%). The highest prevalences of outcomes concerning the total sample were observed in puerperal women who did not practice PA in the third trimester, with 43.9% for cesarean delivery, 7.7% for low birth weight, and 5.5% for premature birth. The odds of cesarean delivery (OR = 1.40; 95%CI 1.10–1.76) and low birth weight (OR = 1.99; 95%CI 1.04–3.79) were, respectively, 40% and 99% higher among puerperal women who did not practice PA in the third trimester of pregnancy when compared to those who practiced PA. There was no association between PA practice and prematurity.

**CONCLUSION:**

Puerperal women who did not practice PA in the third trimester of pregnancy were more likely to have cesarean delivery and low birth weight newborns.

## INTRODUCTION

Physical activity (PA) is recommended for most women during pregnancy with usual risk, i.e., not associated with clinical or obstetric complications^1^. Its practice is associated with favorable health outcomes for both mother and baby^1,2^.

During pregnancy, the regular practice of PA can help prevent several complications, such as gestational diabetes mellitus and systemic arterial hypertension^3^, and provide a reduction in anxiety and postpartum depression, reducing the chances of subsequent complications^4,5^. However, a significant proportion of pregnant women assisted in the Primary Healthcare service report not having time and/or a safe environment for the practice and not receiving adequate advice and recommendations from healthcare professionals regarding its benefits^6^.

In Brazil, the prevalence of women who report practicing PA during pregnancy has shown to be quite diverse, as the methodologies for evaluating this variable are different. In Rio Grande do Sul, the prevalence of PA among leisure-time pregnant women increased between 2004 and 2015. However, the prevalence was still considered low, with only 16% of women practicing PA during the gestational period^7^. In turn, a cohort study with 1,381 women carried out in Maranhão between 2009 and 2010 showed that 60% of pregnant women were physically active^8^.

Among the positive effects of PA on birth and maternal and child health outcomes are the lower chances of cesarean delivery^9^. In 2014, the rate of cesarean sections evaluated in 150 countries reached 18.6%, increasing by 4.4% per year since 1990^12^. In Brazil, the prevalence of cesarean deliveries in 2019 was 55.9%, and even higher (56.8%) in Santa Catarina. Cesarean sections without justified clinical indication have been associated with increased maternal and perinatal morbidity, as women are more exposed to anesthetic complications, internal organ damage, and risk of infections, in addition to other maternal and fetal impairments in subsequent pregnancies^13^.

Another predictor of the newborn’s survival in the short and long term is the birth weight, reflecting the condition of growth in the intrauterine environment during the gestational period^14^. Low birth weight has also shown high prevalence, reaching 16% worldwide and 9.0% in Brazil. Among live births, some are born prematurely and, despite comprising 2% of the total, they represent a large part of child morbidity and mortality^15^. Prematurity and intrauterine growth restriction are among the leading causes of low birth weight. However, unhealthy habits, including a sedentary lifestyle before and during pregnancy, have been identified as potential risk factors for its occurrence^16,17^.

In a systematic review, Dipietro et al.^2^ pointed out that moderate-intensity PA can reduce the risk of some maternal outcomes, such as weight gain and gestational diabetes. However, even with favorable effects, the evidence of its practice for a lower occurrence of cesarean delivery, premature birth, and low birth weight is still limited. Given these gaps in the theme, it is relevant to test the hypothesis that women who practice PA throughout the three trimesters of pregnancy are at lower risk for these negative outcomes during childbirth.

Thus, this study aimed to test the association between PA practice according to the gestational trimesters and the occurrence of cesarean delivery, prematurity, and low birth weight in puerperal women assisted in the Unified Health System (SUS) in Santa Catarina.

## METHODS

This is a cross-sectional study carried out with data from the research entitled “Prenatal and immediate puerperium in Primary Care: evaluation of the management of Rede Cegonha in Santa Catarina.” The research used epidemiology and health assessment tools to diagnose prenatal and postpartum care in the state of Santa Catarina. The estimated population of the state in 2019 was 7,164,788 inhabitants (50.4% female), with 81.5% covered by the Family Health Strategy (ESF) and 91.2% by Primary Care.

The study population consisted of mothers who delivered through the SUS between January and August 2019. The study included hospitals and maternity hospitals that, in 2016, registered 500 or more births and which were located in the nine health macro-regions of the state (Foz do Rio Itajaí, Grande Florianópolis, Extremo Oeste, Centro Oeste, Nordeste, Planalto Norte, Serra Catarinense, Sul, and Vale do Itajaí).

The sample calculation considered a population of 50,000 births/year, a confidence level of 95%, a margin of error of 1.6%, and an estimated prevalence of 50%. In the end, 5% were added, considering losses and refusals. The estimated sample was 3,665 puerperal women, which were proportionally divided into the selected hospitals and maternity hospitals according to the total number of deliveries performed in 2016.

Puerperal women were interviewed in the hospital environment up to 48 hours after delivery. Inclusion criteria were: 1) having lived in Santa Catarina during the entire pregnancy; 2) having performed all prenatal consultations in the SUS or not having had prenatal care; 3) having a child born alive, stillborn, or who died within 48 hours after delivery; and 4) having a child born after the 22^nd^ week of pregnancy and weighing more than 500 grams. Puerperal women who did not want to participate were considered refusals and those who abandoned during the research as losses.

Data collection was performed with the aid of tablets in a structured form on the RedCap platform. Interviewers were previously trained and conducted face-to-face interviews at the birthplace. A reduced questionnaire was applied for data quality control in a random sample (10% of the total) via telephone contact.

The cesarean delivery outcome was self-reported by the interviewees. Premature birth was considered when the gestational age was less than 37 weeks and low birth weight when the newborn weighed less than 2,500 grams. These data were collected from the medical records of the puerperal women, and the information was filled in according to the diagnostic criteria established by the medical team. The practice of PA during any period of pregnancy and in each gestational trimester were the exposures of interest. The interviewees answered yes or no to the following questions: *1) “Apart from your activity at home or work, did you do any type of regular physical exercise during your pregnancy? 2) “Did you do these exercises in the first three months of pregnancy?” 3) Did you do these exercises from the fourth to the sixth month of pregnancy? 4) And in the last three months of pregnancy, did you do these exercises?* Based on the affirmative answers to each question, the prevalence of PA in the periods of interest was estimated.

Sociodemographic control characteristics were self-reported and included age group (≤ 18 years; 19–34 years, and ≥ 35 years), marital status (stable union/with a partner and without a partner), and education (≤ 9 years, 10–12 years, and ≥ 13 years of schooling). Behavioral variables included smoking during pregnancy (yes or no), regular alcohol intake at least once a week during pregnancy (yes or no), body mass index (BMI) before pregnancy (underweight or adequate weight, overweight, obesity), number of previous pregnancies (0 or ≥ 1), and regular physical activity at least twice in the week before pregnancy (yes or no). Finally, maternal health conditions were investigated through the following questions: *“During this pregnancy, did any doctor or health professional say that you had: 1) high-risk pregnancy? 2) gestational diabetes/high blood sugar due to pregnancy?; 3) eclampsia/high blood pressure due to pregnancy?”* Each one of them was analyzed with dichotomized answers (yes or no).

For the analysis of the variables, puerperal women who did not know or did not want to answer the questions were excluded. In the descriptive analysis, absolute and relative frequencies of all variables of interest were estimated. Also, the prevalence and respective 95% confidence intervals (95%CI) of the outcomes according to each exposure of interest. An association between the exposure variables and each outcome was verified using the Multivariate Logistic Regression. From them, crude and adjusted odds ratios and their respective 95% confidence intervals (95%CI) were obtained. The adjustment models were performed separately for each exposure of interest. A single block included all the sociodemographic, behavioral, and maternal health conditions associated with the outcomes in the crude analyses. Data analysis was performed using Stata version 14.1 (StataCorp, Texas, United States), considering a significance level of p < 0.05.

The research was approved by the Ethics Committee for Research with Human Beings (CEPSH) of the Federal University of Santa Catarina (UFSC), under opinion 1,599,464, and wholly followed the ethical precepts recommended by Resolution 510/2016 of the National Health Council.

## RESULTS

The total number of puerperal women analyzed in this study was 3,580 (response rate of 97.7%). The mean age observed was 26.6 years (standard deviation of 6.4 years). The majority reported being married or living with a partner (81.5%), having studied between 10 and 12 years (52.5%), not drinking alcohol (92.8%) or using tobacco during pregnancy (90.7u%), and not having practiced physical activity (PA) before pregnancy (75.4%). Among the health conditions reported, high-risk pregnancy stood out (21.9%), followed by eclampsia (15.8%).

Cesarean delivery was performed in 42.8% of the sample (95%CI 41.2–44.4), with a higher prevalence among those aged over 34 years, more educated, and obese. Premature birth occurred in 7.7% (95%CI 6.8–8.2) of the mothers and was more prevalent among those older and obese, especially among those who had high-risk pregnancies, gestational diabetes, and eclampsia. In turn, low birth weight was found in 5.2% of live newborns (95%CI 4.5–6.4), with higher proportions among those whose mothers were smokers during pregnancy, not had previous pregnancies, with eclampsia, and high-risk pregnancy. Further details are presented in Table 1.

**Table 1:** Description of maternal characteristics according to the prevalence of cesarean delivery, premature birth, and low birth weight in puerperal women assisted by the Unified Health System in the state of Santa Catarina, Brazil, 2020.

Maternal characteristics	n (%)	Cesarean delivery % (95%CI)	Premature birth % (95%CI)	Low birth weight % (95%CI)
Total sample (n = 3,580)				
Age (years) (n = 3,524)				
≤ 18	472 (13.4)	33.5 (29.4–37.9)	7.1 (5.1–9.9)	5.9 (4.1–8.5)
19–34	2,548 (72.3)	43.0 (41.1–44.9)	7.2 (6.2–8.3)	5.0 (4.2–5.9)
≥ 35	504 (14.3)	50.7 (46.3–55.0)	10.7 (8.2–13.8)	5.5 (3.7–7.9)
Marital status (n = 3,576)				
Married/with a partner	2,916 (81.5)	44.0 (42.2–45.8)	7.6 (6.6–8.6)	5.6 (4.8–6.5)
Single/divorced/widow	660 (18.5)	37.5 (33.9–41.3)	8.2 (6.3–10.6)	3.8 (2.5–5.6)
Education (years of study) (n = 3,529)				
≤ 9	1,218 (34.5)	39.6 (36.9–42.4)	7.3 (5.9–8.9)	5.5 (4.4–6.9)
10–12	1,853 (52.5)	42.4 (40.2–44.7)	8.2 (7.1–9.6)	5.0 (4.1–6.1)
≥ 13	458 (13.0)	51.9 (47.3–56.5)	5.9 (3.9–8.5)	4.9 (3.3–7.5)
Smoking during pregnancy (n = 3,555)				
Yes	330 (9.3)	35.1 (30.2–40.5)	9.6 (6.8–13.4)	9.9 (7.0–13.8)
No	3,219 (90.7)	43.6 (41.9–45.3)	7.5 (6.6–8.4)	4.8 (4.1–5.6)
Alcohol use during pregnancy (3,556)				
Yes	255 (7.2)	37.6 (31.9–43.8)	5.9 (3.6–9.6)	4.9 (2.8–8.6)
No	3,301 (92.8)	43.1 (41.5–44.9)	7.9 (7.0–8.9)	5.2 (4.5–6.0)
BMI before pregnancy (n = 3,322)				
Low weight/adequate weight	1,752 (52.7)	37.3 (35.1–39.6)	6.8 (5.7–8.1)	5.5 (4.5–6.7)
Overweight	893 (26.9)	43.9 (40.7–47.3)	7.5 (5.9–9.4)	4.9 (3.6–6.6)
Obesity	677 (20.4)	56.8 (53.0–60.5)	8.8 (6.8–11.2)	3.8 (2.5–5.6)
Number of previous pregnancies (n = 3,579)				
0	1,126 (31.5)	42.2 (39.3–45.1)	6.3 (5.0–7.9)	5.8 (4.5–7.4)
≥ 1	2,453 (68.5)	43.2 (41.2–45.1)	8.3 (7.3–9.5)	4.9 (4.2–5.9)
Physical activity before pregnancy (n = 3,546)				
Yes	872 (24.6)	43.8 (40.5–47.2)	8.9 (7.2–11.0)	5.3 (3.9–7.1)
No	2,674 (75.4)	42.5 (40.6–44.4)	7.4 (6.4–8.4)	5.1 (4.3–6.1)
High-risk pregnancy (n = 3,538)				
Yes	773 (21.9)	56.0 (52.5–59.5)	13.6 (11.4–16.3)	8.3 (6.5–10.6)
No	2,765 (78.1)	39.3 (37.5–41.1)	5.9 (5.1–6.9)	4.4 (3.5–5.2)
Gestational diabetes (n = 3,521)				
Yes	332 (9.4)	56.5 (51.1–61.7)	11.6 (8.6–15.6)	5.7 (3.6–8.9)
No	3,189 (90.6)	41.5 (39.8–43.2)	7.3 (6.5–8.3)	5.1 (4.4–5.9)
Eclampsia (n = 3,552)				
Yes	561 (15.8)	57.5 (53.3–61.5)	13.0 (10.5–16.1)	9.3 (7.1–12.1)
No	2,991 (84.2)	40.2 (38.4–41.9)	6.7 (5.8–7.6)	4.4 (3.7–5.2)
Physical activity during pregnancy (n = 3,573)				
Yes	735 (20.6)	39.9 (36.4–43.5)	7.5 (5.8–9.7)	4.5 (3.3–6.5)
No	2,838 (79.4)	43.5 (41.7–45.4)	7.7 (6.7–8.7)	5.3 (4.6–6.3)
Physical activity - first trimester (n = 3,568)				
Yes	578 (16.2)	41.0 (37.0–45.1)	8.3 (6.3–10.8)	4.2 (2.8–6.3)
No	2,990 (83.8)	43.2 (41.4–44.9)	7.6 (6.7–8.6)	5.4 (4.6–6.3)
Physical activity - second trimester (n = 3,566)				
Yes	548 (15.4)	39.6 (35.6–43.8)	7.9 (5.9–10.5)	4.1 (2.7–6.2)
No	3,018 (84.6)	43.4 (41.6–45.2)	7.6 (6.7–8.6)	5.4 (4.6–6.4)
Physical activity - third trimester (n = 3,567)				
Yes	456 (12.8)	35.5 (31.3–40.0)	7.3 (5.2–10.1)	3.3 (1.9–5.5)
No	3,111 (87.2)	43.9 (42.1–45.6)	7.7 (6.8–8.7)	5.5 (4.7–6.4)

The practice of PA during any period of pregnancy was reported by only 20.6% of puerperal women. Concerning the gestational trimesters, there was a gradual decrease in the prevalence of PA practitioners according to the gestational advance. The value varied, from 16.2% in the first trimester to 15.4% in the second and 12.8% in the third. Considering the total sample, the highest prevalences of cesarean delivery (43.9%), low birth weight (7.7%), and premature birth (5.5%) were observed among mothers who did not practice PA in the third trimester (Table 1).

The adjusted regression models showed that the chance of having a cesarean delivery was 1.40 higher among puerperal women who did not practice PA in the third trimester of pregnancy (OR = 1.40; 95%CI 1.10–1.76), regardless of sociodemographic, behavioral, and health characteristics investigated (Table 2). Similarly, the chances of newborns having low birth weight were 1.99 higher among puerperal women who did not practice PA in the third trimester (OR = 1.99; 95%CI 1.04–3.79), considering the adjusted model (Table 3). For the occurrence of premature birth, no statistically significant odds ratios were observed in the analysis models, indicating that the practice of PA was not associated with this outcome (Table 4).

**Table 2:** Multivariate Logistic Regression analysis models between the practice of physical activity (PA) in the gestational trimesters and the occurrence of cesarean delivery in puerperal women assisted by the Unified Health System in Santa Catarina, Brazil, 2020.

	Crude analysis OR (95%CI)	Adjusted analysis^a^ OR (95%CI)
PA during pregnancy		
Yes	1.00	1.00
No	1.16 (0.99–1.37)	1.20 (0.98–1.46)
PA - first trimester		
Yes	1.00	1.00
No	1.09 (0.91–1.31)	1.16 (0.93–1.44)
PA - second trimester		
Yes	1.00	1.00
No	1.17 (0.97–1.40)	1.24 (0.99–1.55)
PA - third trimester		
Yes	1.00	1.00
No	1.41 (1.15–1.74)^b^	1.40 (1.10–1.76)^b^

OR: odds ratio; 95%CI: 95% confidence interval.

^a^ Adjustment for age group, marital status, education, smoking and alcohol during pregnancy, number of previous pregnancies, body mass index, PA before pregnancy, high-risk pregnancy, gestational diabetes, and eclampsia.

^b^ Statistical significance (p ≤ 0.05).

**Table 3:** Multivariate Logistic Regression analysis models between the practice of physical activity (PA) in the gestational trimesters and the occurrence of low birth weight in puerperal women assisted by the Unified Health System in Santa Catarina, Brazil, 2020.

	Crude analysis OR (95%CI)	Adjusted analysis^a^ OR (95%CI)
PA during pregnancy		
Yes	1.00	1.00
No	1.16 (0.78–1.72)	1.34 (0.83–2.15)
PA - first trimester		
Yes	1.00	1.00
No	1.28 (0.82–2.01)	1.74 (0.99–3.05)
PA - second trimester		
Yes	1.00	1.00
No	1.35 (0.85–2.14)	1.49 (0.86–2.59)
PA - third trimester		
Yes	1.00	1.00
No	1.70 (0.98–2.96)	1.99 (1.04–3.79)^b^

OR: odds ratio; IC95%: 95% confidence interval.

^a^ Adjustment for age group, marital status, education, smoking and alcohol during pregnancy, number of previous pregnancies, body mass index, PA before pregnancy, high-risk pregnancy, gestational diabetes, and eclampsia.

^b^ Statistical significance (p ≤ 0.05).

**Table 4:** Multivariate Logistic Regression analysis models between the practice of physical activity (PA) in the gestational trimesters and the occurrence of premature birth in puerperal women assisted by the Unified Health System in the state of Santa Catarina, Brazil, 2020.

	Crude analysis OR (95%CI)	Adjusted analysis^a^ OR (95%CI)
PA during pregnancy		
Yes	1.00	1.00
No	1.02 (0.75–1.38)	1.15 (0.78–1.69)
PA - first trimester		
Yes	1.00	1.00
No	0.91 (0.65–1.26)	1.07 (0.71–1.62)
PA - second trimester		
Yes	1.00	1.00
No	0.96 (0.68–1.34)	1.08 (0.71–1.63)
PA - third trimester		
Yes	1.00	1.00
No	1.06 (0.73–1.55)	1.12 (0.72–1.74)

OR: odds ratio; IC95%: 95% confidence interval.

^a^ Adjustment for age group, marital status, education, smoking and alcohol during pregnancy, number of previous pregnancies, body mass index, PA before pregnancy, high-risk pregnancy, gestational diabetes, and eclampsia.

## DISCUSSION

The main results of the present study showed that puerperal women who did not practice PA during the third gestational trimester were more likely to undergo cesarean delivery and have low birth weight newborns. On the other hand, PA practice was not significantly associated with prematurity regardless of the gestational period.

The prevalence of pregnant women practicing PA at any time of pregnancy observed in the present study (20.6%) was found to be intermediate to those found in pregnant women evaluated in the cities of Rio Grande (RS) (32.8%)^11^ and Pelotas (RS) (12.9%)^4^. In turn, Regô et al.^8^ observed that 60.3% of the pregnant women participating in the BRISA cohort in São Luís (MA) were physically active; however, considering only the PA practiced during leisure and evaluated in the second gestational trimester. In any case, direct comparisons become limited due to methodological differences in measuring PA data between studies.

Some studies also support our finding that there is a gradual decrease in PA practice as the pregnancy progresses. Watson et al.^18^ showed a reduction in the level of PA from the second to the third gestational trimester (12.8% to 9.7%). Ko et al.^19^ also showed that the mean self-reported PA using a visual analog scale was higher in the second trimester (5.01 ± 1.99), lower in the third (4.62 ± 1.97). In the first trimesters, pregnant women are believed to feel more confident performing PA, as they are more concerned about the baby’s health and weight gain. At the end of pregnancy, it is common to experience excessive discomfort and some contraindications for physical exercise, which may justify the abandonment of regular practice due to weight gain^20^. Pre-gestational physical inactivity combined with excessive weight gain in the last months of pregnancy and social models concerning the physical behavior acquired during childhood are among the factors that lead to a decrease in PA practice during the gestational period^21^.

In the present study, puerperal women who did not practice PA during the third gestational trimester had greater chances of having a cesarean delivery. A study conducted in Taiwan found that the practice of PA in the third trimester differed significantly between women who underwent unplanned cesarean delivery and those who had a vaginal delivery, with greater chances of cesarean delivery for women who had low levels of PA in the third trimester^19^. A study with a population of nulliparous women residing in Denmark found that the increasing PA level during leisure time was associated with a less complicated mode of delivery, i.e., with a lower probability of performing emergency cesarean delivery or vacuum extraction. The authors suggest that leisure-time PA until the end of pregnancy may play an essential role in reducing emergency cesarean sections and assisted vaginal deliveries. This is of great public health interest since PA is healthy behavior, accessible, and causes little or no negative effect on pregnant women^22^. Rajabi et al.^23^ also showed lower odds (OR = 0.68; 95%CI 0.47–0.97) of having a cesarean delivery in women who increased their PA level during pregnancy compared to the pre-pregnancy period.

Even without considering the assessment of PA by gestational trimesters, a meta-analysis carried out with 12 studies concluded that regular physical exercise at low or moderate levels during pregnancy seems to modestly increase the chance of vaginal delivery in healthy pregnant women^3^. In the present study, PA practice during any pregnancy period, when adjusted only for sociodemographic and behavioral characteristics and without considering the risk conditions for maternal health, also reduced the chances of cesarean delivery. Supporting these findings, Chia-Hui Chen et al.^24^ showed that total PA, unrelated to trimesters, increased the chances of vaginal delivery (AOR = 1.017; p < 0.05). Furthermore, the practice of a structured PA program by pregnant women has been observed as being able to reduce the risk of cesarean sections by 15%, highlighting the importance of pregnant women being encouraged to exercise following the recommendations of the American College of Obstetricians and Gynecologists (ACOG)^25^.

In the present study, not practicing PA during the third trimester of pregnancy was also significantly associated with greater chances of low birth weight. However, to date, few studies in the literature specifically tested this association in the third trimester of pregnancy. In the findings of Clapp et al.^26^, pregnant women who practiced PA from the 8^th^ week of gestation, for 20 minutes and 3 to 5 times a week, gave birth to heavier newborns when compared to sedentary pregnant women (3,750 g–3,490 g). Regô et al.^8^ only evaluated PA practiced in the second gestational trimester. No association was observed concerning low weight, regardless of the PA level of the pregnant women. Other population studies carried out with large Norwegian^27^ and Danish^28^ women samples also did not identify an association between PA practice during pregnancy and low birth weight. On the other hand, they observed that the practice of PA 3 to 5 times a week during the second and third trimesters proved to be protective (OR = 0.82; 95%CI 0.73–0.91) concerning prematurity^27^. This study did not show significant associations between the practice of PA (regardless of the gestational trimester) and the lower occurrence of prematurity. However, it is noteworthy that these are commonly related outcomes, as babies born preterm also tend to have low birth weight.

Haakstad et al.^21^ evaluated pregnant women in the 2^nd^ and 3^rd^ trimesters of pregnancy who practiced aerobic exercise twice a week for 60 minutes for 12 weeks. The findings did not show statistically significant differences for mean and low birth weight (< 2,500 g), and regular PA during pregnancy did not cause prematurity. In a more recent study, Beetham et al.^29^ observed through a meta-analysis that women who practice vigorous PA in any gestational period were protected against low birth weight through relative risk (RR = 0.44) and prematurity (RR = 0.20). However, it is worth considering that the physiological explanations for PA practice during pregnancy and the occurrence of premature births are still controversial. On the one hand, it is believed that the stimulus to premature birth may occur due to the larger release of catecholamines and norepinephrine. This release results from physical exercise, inducing myometrial activity and accelerates labor^10^. Moreover, on the other hand, the fact that PA reduces oxidative stress and improves placental vascularization could reduce the risk of premature births. Also, it is associated with a reduction in the incidence of gestational diabetes mellitus and hypertensive disorders^10^.

Given these findings, despite the scarcity of studies supporting the positive effects of PA practice in each trimester for the favorable outcomes of the puerperium, the importance of engaging in PA is considered quite evident, especially at the end of pregnancy. Regular physical exercise is recommended for healthy pregnant women, both for athletes (considering training adaptations) and those who were not physically active in the pre-pregnancy period, who should follow an exercise program with a gradual increase in intensity during pregnancy. In addition to all the benefits related to the newborn, the practice of regular PA during pregnancy can promote the general well-being of women, help maintain adequate weight gain, reduce hypertensive disorders, risk of pre-eclampsia, deep vein thrombosis, and gestational diabetes^30^.

To better understand the relationship between PA practice during gestational periods and the outcomes in the puerperium, knowing other factors such as intensity, duration, frequency, and type of physical exercise is also considered essential. These aspects can be pointed out as a limitation of the present study since the evaluated puerperal women were not asked about these characteristics. Our findings indicate the importance of gestational PA practice (especially in the third trimester) to achieve better outcomes in childbirth. However, it is noteworthy that some women may have had absolute contraindications for its performance, which may not have been identified in our analyses. Furthermore, the information was subjective, which may have underestimated or overestimated the level of PA reported as a result of perception and memory. Finally, it is understood that if the analyses had not been carried out according to the state’s macro-regions, they could reflect cultural, geographic, public policy implementation disparities and enable more specific results for the actions of local healthcare managers.

However, the study was carried out with a probabilistic sample of puerperal women from all over the state of Santa Catarina and who underwent childbirth and prenatal care exclusively through the Unified Health System. As they are usually evaluated together with samples from maternity hospitals in the private network, this joint analysis should be considered a positive study point. According to the gestational trimester, PA assessment is also noteworthy. The findings strengthen and support PA early in pregnancy while also denoting the importance of carrying it out until delivery. Another relevant point is that the present study uses empirical data on PA during pregnancy, one of the leading health behaviors that can be modified and can subsidize public actions and policies to improve prenatal care.

Even with the relevance of our findings, it is worth noting that just over a fifth of the sample (20.6%) reported practicing PA during any gestational period. It indicates that women who remain active during pregnancy still represent a minority among all who perform prenatal care in the public network of Santa Catarina. Thus, the theme’s relevance to encourage PA practice during pregnancy is denoted, often neglected by healthcare professionals and services. Promoting new studies with this audience is also considered essential, which aims to identify the main factors related to supply, access, and attitudes that facilitate or hinder the adoption of active and healthy behaviors during pregnancy.

Thus, not practicing PA in the third trimester of pregnancy is concluded as increasing the chances of cesarean delivery and low birth weight. These results suggest the importance of encouraging the practice of PA by strengthening public policies involving pregnant women through information and care in the system, providing a reduction in public hospital expenses, and providing a better quality of life for the pregnant woman and the baby.
