# Supplementary material for: Physical activity in gestational trimesters and perinatal outcomes in SUS puerperal women
Source: Rev Saude Publica. 2021 Oct 13;55:58. doi: 10.11606/s1518-8787.2021055003067 (PMC8522735; doi:10.11606/s1518-8787.2021055003067)
Supplement: Translation

# Translation: Atividade física nos trimestres gestacionais e desfechos perinatais em puérperas do SUS

**Authors:** Tauana Prestes Schmidt, Talita Tuon, Katia J. Pudla Wagner, Antonio Fernando Boing, Ana Lúcia Danielewicz

**Affiliations:** I Brasil Universidade Federal de Santa Catarina. Programa de Pós-Graduação em Ciências da Reabilitação. Araranguá, SC, Brasil; II Brasil Universidade Federal de Santa Catarina. Coordenadoria Especial de Biociências e Saúde Única. Curitibanos, SC, Brasil; III Brasil Universidade Federal de Santa Catarina. Departamento de Saúde Pública. Florianópolis, SC, Brasil; IV Brasil Universidade Federal de Santa Catarina Departamento de Ciências da Saúde. Araranguá, SC, Brasil

**Keywords:** Gestantes, Exercício Físico, Parto Normal, Cesárea, Nascimento Prematuro, Estudos Transversais

## Abstract

**OBJETIVO:**

Testar a associação entre a prática de atividade física (AF) de acordo com os trimestres gestacionais e a ocorrência de parto cesáreo, prematuridade e baixo peso ao nascer em puérperas atendidas no Sistema Único de Saúde de Santa Catarina, Brasil.

**MÉTODOS:**

Estudo transversal realizado com amostra probabilística de puérperas que tiveram seus partos em maternidades da rede pública de Santa Catarina no período de janeiro a agosto de 2019. O desfecho parto cesáreo foi autorreferido e as informações sobre parto prematuro (< 37 semanas gestacionais) e baixo peso ao nascer (< 2.500 gramas) foram obtidas dos prontuários. A prática de AF durante a gestação e conforme cada trimestre foi autorreferida. Foram realizadas análises de Regressão Logística Multivariável e entrevistas com 3.580 puérperas.

**RESULTADOS:**

A prática de AF durante qualquer período da gestação foi relatada por 20,6% da amostra, com redução gradativa das prevalências conforme os trimestres gestacionais (16,2%, 15,4% e 12,8%). As maiores prevalências dos desfechos em relação à amostra total, foram observadas nas puérperas não praticantes de AF no terceiro trimestre, sendo 43,9% para o parto cesáreo, 7,7% para o baixo peso ao nascer e 5,5% para o parto prematuro. As chances de parto cesáreo (OR = 1,40; IC95% 1,10–1,76) e de baixo peso ao nascer (OR = 1,99; IC95% 1,04–3,79) foram, respectivamente, 40% e 99% maiores entre as puérperas que não praticaram AF no terceiro trimestre da gestação quando comparadas àquelas que praticaram AF. Não houve associação da prática de AF com a prematuridade.

**CONCLUSÃO:**

As puérperas que não praticavam AF no terceiro trimestre da gestação tiveram maiores chances de parto cesáreo e de terem recém-nascidos com baixo peso.

## INTRODUÇÃO

A atividade física (AF) é recomendada para a maioria das mulheres durante a gestação de risco habitual, ou seja, que não esteja associada a complicações clínicas ou obstétricas^1^. Sua prática está associada a desfechos favoráveis à saúde tanto para a mãe quanto para o bebê^1,2^.

A prática regular de AF durante a gestação pode auxiliar na prevenção de diversas complicações, como a diabetes mellitus gestacional e a hipertensão arterial sistêmica^3^, bem como propiciar redução da ansiedade e depressão pós-parto, reduzindo as chances de complicações subsequentes^4,5^. No entanto, importante proporção de gestantes atendidas no serviço de Atenção Primária à Saúde refere não ter tempo e/ou ambiente seguro para a prática, assim como não recebe aconselhamentos e recomendações adequadas dos profissionais de saúde em relação aos seus benefícios^6^.

No Brasil, a prevalência de mulheres que relatam praticar AF na gestação tem se mostrado bastante diversificada, visto que as metodologias para avaliação dessa variável são distintas. No Rio Grande do Sul, a prevalência de AF entre gestantes no lazer aumentou entre os anos de 2004 e 2015. Porém, a prevalência ainda foi considerada baixa, com apenas 16% das mulheres praticando AF no período gestacional^7^. Por sua vez, um estudo de coorte com 1.381 mulheres realizado no Maranhão entre 2009 e 2010 mostrou que 60% das gestantes eram fisicamente ativas^8^.

Dentre os efeitos positivos da AF sobre os desfechos de nascimento e de saúde materno-infantil estão as menores chances de parto cesáreo^9^. Em 2014, a taxa de cesarianas avaliada em 150 países chegou a 18,6%, com aumento de 4,4% ao ano desde 1990^12^. No Brasil, a prevalência de partos cesáreos em 2019 foi de 55,9%, sendo ainda maior (56,8%) no estado de Santa Catarina. As cesarianas sem indicação clínicas justificadas têm sido associadas ao aumento da morbidade materna e perinatal, visto que as mulheres ficam mais expostas às complicações anestésicas, lesões de órgãos internos e risco de infecções, além de outros comprometimentos maternos e fetais nas gestações subsequentes^13^.

Outro preditor da sobrevivência do recém-nascido a curto e longo prazo é o seu peso no nascimento, refletindo a condição de crescimento no ambiente intrauterino durante o período gestacional^14^. O baixo peso ao nascer também tem apresentado prevalências elevadas, chegando a 16% no mundo e a 9,0% no Brasil. Dentre os nascidos vivos, há aqueles que nascem prematuros e que, apesar de compreenderem 2% do total, representam grande parte da morbimortalidade infantil^15^. A prematuridade e a restrição do crescimento intrauterino estão entre os principais causadores do baixo peso ao nascer. Porém, os hábitos não saudáveis, incluindo o sedentarismo antes e durante a gestação, têm sido apontados como fatores de risco potenciais para sua ocorrência^16,17^.

Em revisão sistemática, Dipietro et al.^2^ apontaram que a AF de intensidade moderada é capaz de reduzir o risco de alguns desfechos maternos, tais como o ganho de peso e a diabetes gestacional. Porém, mesmo com efeitos favoráveis, ainda são limitadas as evidências da sua prática para a menor ocorrência de parto cesáreo, nascimento prematuro e baixo peso ao nascer. Diante de tais lacunas sobre a temática, torna-se relevante testar a hipótese de que as mulheres que praticam AF ao longo dos três trimestres da gestação apresentam menores riscos para a ocorrência desses desfechos negativos no parto.

Sendo assim, o objetivo deste estudo foi testar a associação entre a prática de AF de acordo com os trimestres gestacionais e a ocorrência de parto cesáreo, prematuridade e baixo peso ao nascer em puérperas atendidas no Sistema Único de Saúde (SUS) em de Santa Catarina.

## MÉTODOS

Trata-se de um estudo transversal realizado com dados da pesquisa intitulada “Pré-Natal e puerpério imediato na Atenção Básica: avaliação da gestão da Rede Cegonha em Santa Catarina”. A pesquisa utilizou ferramentas da epidemiologia e da avaliação em saúde para realizar um diagnóstico da atenção ao pré-natal e pós-parto no estado de Santa Catarina. A população estimada do estado em 2019 era de 7.164.788 habitantes (50,4% do sexo feminino), sendo 81,5% cobertos pela Estratégia Saúde da Família (ESF) e 91,2% pela Atenção Primária.

A população de estudo foi composta pelas puérperas que realizaram seus partos pelo SUS entre os meses de janeiro a agosto de 2019. Foram incluídos no estudo os hospitais e maternidades que, em 2016, registraram 500 ou mais partos e que estavam localizados nas nove macrorregiões de saúde do estado (Foz do Rio Itajaí, Grande Florianópolis, Grande Oeste, Meio Oeste, Nordeste, Planalto Norte, Serra Catarinense, Sul e Vale do Itajaí).

O cálculo amostral considerou população de 50.000 nascimentos/ano, nível de confiança de 95%, margem de erro de 1,6% e prevalência estimada de 50%. Ao final, foram somados 5%, considerando-se as perdas e as recusas. A amostra estimada foi de 3.665 puérperas, as quais foram divididas proporcionalmente nos hospitais e maternidades selecionados conforme o total de partos realizados em 2016.

As puérperas foram entrevistadas no ambiente hospitalar até 48 horas após o parto. Os critérios de inclusão foram: 1) ter residido em Santa Catarina durante toda a gestação; 2) ter realizado todas as consultas do pré-natal no SUS ou não ter realizado pré-natal; 3) ter tido filho nascido vivo, natimorto ou com óbito em até 48 horas pós-parto; e 4) ter tido filho nascido a partir da 22ª semana de gestação e pesando mais de 500 gramas. As puérperas que não quiseram participar foram consideradas como recusas e aquelas que abandonaram no decorrer da pesquisa como perdas.

A coleta dos dados foi realizada com o auxílio de *tablets* em formulário estruturado na plataforma RedCap. Os entrevistadores foram previamente treinados e realizaram entrevistas face a face no local do parto. Para o controle de qualidade dos dados aplicou-se questionário reduzido em amostra aleatória (10% do total) via contato telefônico.

O desfecho parto cesáreo foi autorreferido pelas entrevistadas. O parto prematuro foi considerado quando a idade gestacional foi menor que 37 semanas e o baixo peso ao nascer quando o recém-nascido pesou menos que 2.500 gramas. Esses dados foram coletados dos prontuários das puérperas, sendo as informações preenchidas de acordo com os critérios diagnósticos estabelecidos pela equipe médica. A prática de AF durante qualquer período da gestação e em cada trimestre gestacional foram as exposições de interesse. As entrevistadas responderam sim ou não às seguintes questões: *1) “Sem contar sua atividade em casa ou no emprego, a Sra. fazia algum tipo de exercício físico regular durante a gravidez? 2) “A Sra. fez estes exercícios nos primeiros três meses, de gravidez?” 3) A Sra. fez estes exercícios do quarto ao sexto mês de gravidez? 4) E nos últimos três meses de gravidez, a Sra. fez estes exercícios?* Com base nas respostas afirmativas de cada questão foram estimadas as prevalências de AF dos períodos de interesse.

As características de controle sociodemográficas foram autorreferidas e incluíram a faixa etária (≤ 18 anos; 19–34 anos e ≥ 35 anos), estado civil (união estável/com companheiro e sem companheiro) e escolaridade (≤ 9 anos de estudo, 10–12 anos de estudo e ≥ 13 anos de estudo). As variáveis comportamentais incluíram o tabagismo durante a gestação (sim ou não); ingestão de bebida alcoólica regular em pelo menos uma vez na semana durante a gestação (sim ou não); índice de massa corporal (IMC) antes da gestação (baixo peso ou peso adequado; sobrepeso; obesidade); número de gestações prévias (0 ou ≥ 1) e prática de atividade física regular por pelo menos duas vezes na semana antes da gestação (sim ou não). Por fim, as condições de saúde materna foram investigadas por meio das seguintes questões: *“Durante esta gravidez, algum médico ou profissional de saúde disse que a Sra. tinha: 1) gravidez de alto risco? 2) diabetes gestacional/açúcar alto no sangue por causa da gravidez?; 3) eclampsia/pressão alta devido a gravidez?”* Cada uma delas foi analisada com as respostas dicotomizadas (sim ou não).

Para as análises das variáveis, foram excluídas as puérperas que não sabiam ou não quiseram responder as questões realizadas. Na análise descritiva foram estimadas as frequências absolutas e relativas de todas as variáveis de interesse, bem como as prevalências e respectivos intervalos de confiança de 95% (IC95%) dos desfechos conforme cada exposição de interesse. A existência de associação entre as variáveis de exposição e cada desfecho foi verificada por meio da Regressão Logística Multivariável. A partir delas foram obtidas as razões de chances (*odds ratio*) brutas e ajustadas e seus respectivos intervalos de confiança 95% (IC95%). Os modelos de ajuste foram realizados separadamente para cada exposição de interesse e incluíram em único bloco todas as características sociodemográficas, comportamentais e condições da saúde materna que se mostraram associadas aos desfechos nas análises brutas. A análise dos dados foi realizada no programa Stata versão 14.1 (StataCorp, Texas, Estados Unidos), considerando nível de significância de p < 0,05.

A pesquisa recebeu aprovação pelo Comitê de Ética em Pesquisa com Seres Humanos (CEPSH) da Universidade Federal de Santa Catarina (UFSC), sob o parecer 1.599.464, e seguiu integralmente os preceitos éticos preconizados pela Resolução 510/2016 do Conselho Nacional de Saúde.

## RESULTADOS

O total de puérperas analisadas no presente estudo foi de 3.580 (taxa de resposta de 97,7%). A média de idade observada foi de 26,6 anos (desvio padrão de 6,4 anos). A maioria relatou estar casada ou viver com companheiro (81,5%), ter estudado entre 10 e 12 anos (52,5%), não ter ingerido álcool (92,8%) ou utilizado tabaco durante a gestação (90,7%) e não ter praticado atividade física (AF) antes da gestação (75,4%). Dentre as condições de saúde relatadas, destacou-se a gestação de alto risco (21,9%), seguida da eclampsia (15,8%).

O parto cesáreo foi realizado em 42,8% da amostra (IC95% 41,2–44,4), com maior prevalência entre aquelas com idade acima de 34 anos, maior escolaridade e obesas. O parto prematuro ocorreu em 7,7% (IC95% 6,8–8,2) das puérperas e se mostrou mais prevalente entre as que tinham maior idade e eram obesas, porém, com destaque para aquelas que tiveram gestação de alto risco, diabetes gestacional e eclampsia. Por sua vez, com o grupo de baixo peso ao nascer foi verificado em 5,2% dos recém-nascidos vivos (IC95% 4,5–6,4), com maiores proporções entre aqueles cujas mães foram fumantes durante a gestação, não tiveram gestações prévias, com eclampsia e gestação de alto risco. Maiores detalhes estão apresentados na Tabela 1.

**Tabela 1:** Descrição das características maternas de acordo com as prevalências de parto cesáreo, parto prematuro e baixo peso ao nascer nas puérperas atendidas no Sistema Único de Saúde no estado de Santa Catarina, Brasil, 2020.

Características maternas	n (%)	Parto cesáreo % (IC95%)	Parto prematuro % (IC95%)	Baixo peso ao nascer % (IC95%)
Amostra total (n = 3.580)				
Idade (anos) (n = 3.524)				
≤ 18	472 (13,4)	33,5 (29,4–37,9)	7,1 (5,1–9,9)	5,9 (4,1–8,5)
19–34	2.548 (72,3)	43,0 (41,1–44,9)	7,2 (6,2–8,3)	5,0 (4,2–5,9)
≥ 35	504 (14,3)	50,7 (46,3–55,0)	10,7 (8,2–13,8)	5,5 (3,7–7,9)
Estado civil (n = 3.576)				
Casada/com companheiro	2.916 (81,5)	44,0 (42,2–45,8)	7,6 (6,6–8,6)	5,6 (4,8–6,5)
Solteira/divorciada/viúva	660 (18,5)	37,5 (33,9–41,3)	8,2 (6,3–10,6)	3,8 (2,5–5,6)
Escolaridade (anos de estudo) (n = 3.529)				
≤ 9	1.218 (34,5)	39,6 (36,9–42,4)	7,3 (5,9–8,9)	5,5 (4,4–6,9)
10–12	1.853 (52,5)	42,4 (40,2–44,7)	8,2 (7,1–9,6)	5,0 (4,1–6,1)
≥ 13	458 (13,0)	51,9 (47,3–56,5)	5,9 (3,9–8,5)	4,9 (3,3–7,5)
Fumo durante a gestação (n = 3.555)				
Sim	330 (9,3)	35,1 (30,2–40,5)	9,6 (6,8–13,4)	9,9 (7,0–13,8)
Não	3.219 (90,7)	43,6 (41,9–45,3)	7,5 (6,6–8,4)	4,8 (4,1–5,6)
Uso de álcool durante a gestação (3.556)				
Sim	255 (7,2)	37,6 (31,9–43,8)	5,9 (3,6–9,6)	4,9 (2,8–8,6)
Não	3.301 (92,8)	43,1 (41,5–44,9)	7,9 (7,0–8,9)	5,2 (4,5–6,0)
IMC antes da gestação (n = 3.322)				
Baixo peso/peso adequado	1.752 (52,7)	37,3 (35,1–39,6)	6,8 (5,7–8,1)	5,5 (4,5–6,7)
Excesso de peso	893 (26,9)	43,9 (40,7–47,3)	7,5 (5,9–9,4)	4,9 (3,6–6,6)
Obesidade	677 (20,4)	56,8 (53,0–60,5)	8,8 (6,8–11,2)	3,8 (2,5–5,6)
Número de gestações anteriores (n = 3.579)				
0	1.126 (31,5)	42,2 (39,3–45,1)	6,3 (5,0–7,9)	5,8 (4,5–7,4)
≥ 1	2.453 (68,5)	43,2 (41,2–45,1)	8,3 (7,3–9,5)	4,9 (4,2–5,9)
Atividade física antes da gestação (n = 3.546)				
Sim	872 (24,6)	43,8 (40,5–47,2)	8,9 (7,2–11,0)	5,3 (3,9–7,1)
Não	2.674 (75,4)	42,5 (40,6–44,4)	7,4 (6,4–8,4)	5,1 (4,3–6,1)
Gestação de alto risco (n = 3.538)				
Sim	773 (21,9)	56,0 (52,5–59,5)	13,6 (11,4–16,3)	8,3 (6,5–10,6)
Não	2.765 (78,1)	39,3 (37,5–41,1)	5,9 (5,1–6,9)	4,4 (3,5–5,2)
Diabetes gestacional (n = 3.521)				
Sim	332 (9,4)	56,5 (51,1–61,7)	11,6 (8,6–15,6)	5,7 (3,6–8,9)
Não	3.189 (90,6)	41,5 (39,8–43,2)	7,3 (6,5–8,3)	5,1 (4,4–5,9)
Eclâmpsia (n = 3.552)				
Sim	561 (15,8)	57,5 (53,3–61,5)	13,0 (10,5–16,1)	9,3 (7,1–12,1)
Não	2.991 (84,2)	40,2 (38,4–41,9)	6,7 (5,8–7,6)	4,4 (3,7–5,2)
Atividade física durante a gestação (n = 3.573)				
Sim	735 (20,6)	39,9 (36,4–43,5)	7,5 (5,8–9,7)	4,5 (3,3–6,5)
Não	2.838 (79,4)	43,5 (41,7–45,4)	7,7 (6,7–8,7)	5,3 (4,6–6,3)
Atividade física primeiro trimestre (n = 3.568)				
Sim	578 (16,2)	41,0 (37,0–45,1)	8,3 (6,3–10,8)	4,2 (2,8–6,3)
Não	2.990 (83,8)	43,2 (41,4–44,9)	7,6 (6,7–8,6)	5,4 (4,6–6,3)
Atividade física segundo trimestre (n = 3.566)				
Sim	548 (15,4)	39,6 (35,6–43,8)	7,9 (5,9–10,5)	4,1 (2,7–6,2)
Não	3.018 (84,6)	43,4 (41,6–45,2)	7,6 (6,7–8,6)	5,4 (4,6–6,4)
Atividade física terceiro trimestre (n = 3.567)				
Sim	456 (12,8)	35,5 (31,3–40,0)	7,3 (5,2–10,1)	3,3 (1,9–5,5)
Não	3.111 (87,2)	43,9 (42,1–45,6)	7,7 (6,8–8,7)	5,5 (4,7–6,4)

A prática de AF durante qualquer período da gestação foi relatada por apenas 20,6% das puérperas. Em relação aos trimestres gestacionais, verificou-se diminuição gradativa da prevalência de praticantes de AF conforme o avanço gestacional, passando de 16,2% no primeiro trimestre para 15,4% no segundo e 12,8% no terceiro. Considerando-se a amostra total, as maiores prevalências de parto cesáreo (43,9%), baixo peso ao nascer (7,7%) e parto prematuro (5,5%) foram observadas entre as puérperas que não praticaram AF no terceiro trimestre (Tabela 1).

Os modelos ajustados de regressão mostraram que a chance de ter parto cesáreo foi 1,40 maior entre as puérperas que não praticaram AF no terceiro trimestre da gestação (OR = 1,40; IC95% 1,10–1,76), independente das características sociodemográficas, comportamentais e de saúde investigadas (Tabela 2). De modo semelhante, as chances de os recém-nascidos apresentarem baixo peso ao nascer foi 1,99 maior entre as puérperas não praticantes de AF no terceiro trimestre (OR = 1,99; IC95% 1,04–3,79), considerando o modelo ajustado (Tabela 3). Para a ocorrência de parto prematuro não foram observadas razões de chances estatisticamente significativas nos modelos de análise, indicando que a prática de AF não se mostrou associada a esse desfecho (Tabela 4).

**Tabela 2:** Modelos de análise de Regressão Logística Multivariável entre a prática de atividade física (AF) nos trimestres gestacionais e a ocorrência de parto cesáreo nas puérperas atendidas no Sistema Único de Saúde no estado de Santa Catarina, Brasil, 2020.

	Análise bruta OR (IC95%)	Análise ajustada^a^ OR (IC95%)
AF durante a gestação		
Sim	1,00	1,00
Não	1,16 (0,99–1,37)	1,20 (0,98–1,46)
AF primeiro trimestre		
Sim	1,00	1,00
Não	1,09 (0,91–1,31)	1,16 (0,93–1,44)
AF segundo trimestre		
Sim	1,00	1,00
Não	1,17 (0,97–1,40)	1,24 (0,99–1,55)
AF terceiro trimestre		
Sim	1,00	1,00
Não	1,41 (1,15–1,74)^b^	1,40 (1,10–1,76)^b^

OR: *odds ratio*; IC95%: Intervalo de confiança de 95%.

^a^ Ajuste para faixa etária, estado civil, escolaridade, fumo e álcool durante a gestação, número de gestações prévias, índice de massa corporal, AF antes da gestação, gestação de alto risco, diabetes gestacional e eclampsia.

^b^ Significância estatística (p ≤ 0,05).

**Tabela 3:** Modelos de análise de Regressão Logística Multivariável entre a prática de atividade física (AF) nos trimestres gestacionais e a ocorrência de baixo peso ao nascer nas puérperas atendidas no Sistema Único de Saúde no estado de Santa Catarina, Brasil, 2020.

	Análise bruta OR (IC95%)	Análise ajustada^a^ OR (IC95%)
AF durante a gestação		
Sim	1,00	1,00
Não	1,16 (0,78–1,72)	1,34 (0,83–2,15)
AF primeiro trimestre		
Sim	1,00	1,00
Não	1,28 (0,82–2,01)	1,74 (0,99–3,05)
AF segundo trimestre		
Sim	1,00	1,00
Não	1,35 (0,85–2,14)	1,49 (0,86–2,59)
AF terceiro trimestre		
Sim	1,00	1,00
Não	1,70 (0,98–2,96)	1,99 (1,04–3,79)^b^

OR: *odds ratio*; IC95%: Intervalo de confiança de 95%.

^a^ Ajuste para faixa etária, estado civil, escolaridade, fumo e álcool durante a gestação, número de gestações prévias, índice de massa corporal, AF antes da gestação, gestação de alto risco, diabetes gestacional e eclampsia.

^b^ Significância estatística (p ≤ 0,05).

**Tabela 4:** Modelos de análise de Regressão Logística Multivariável entre a prática de atividade física (AF) nos trimestres gestacionais e a ocorrência de parto prematuro nas puérperas atendidas no Sistema Único de Saúde no estado de Santa Catarina, Brasil, 2020.

	Análise bruta OR (IC95%)	Análise ajustada^a^ OR (IC95%)
AF durante a gestação		
Sim	1,00	1,00
Não	1,02 (0,75–1,38)	1,15 (0,78–1,69)
AF primeiro trimestre		
Sim	1,00	1,00
Não	0,91 (0,65–1,26)	1,07 (0,71–1,62)
AF segundo trimestre		
Sim	1,00	1,00
Não	0,96 (0,68–1,34)	1,08 (0,71–1,63)
AF terceiro trimestre		
Sim	1,00	1,00
Não	1,06 (0,73–1,55)	1,12 (0,72–1,74)

OR: *odds ratio*; IC95%: Intervalo de confiança de 95%.

^a^ Ajuste para faixa etária, estado civil, escolaridade, fumo e álcool durante a gestação, número de gestações prévias, índice de massa corporal, AF antes da gestação, gestação de alto risco, diabetes gestacional e eclampsia.

## DISCUSSÃO

Os principais resultados do presente estudo mostraram que as puérperas que não praticavam AF durante o terceiro trimestre gestacional tiveram maiores chances de realizar parto cesáreo e de terem recém-nascidos com baixo peso. Por outro lado, observou-se que a prática de AF, independente do período gestacional, não se associou significativamente à ocorrência de prematuridade.

Com relação à prevalência de gestantes praticantes de AF em qualquer período da gestação, verificou-se que a observada no presente estudo (20,6%) se mostrou intermediária às encontradas nas gestantes avaliadas nos municípios de Rio Grande (RS) (32,8%)^11^ e Pelotas (RS) (12,9%)^4^. Já o estudo de Regô et al.^8^ observou que 60,3% das gestantes participantes da coorte BRISA em São Luís (MA) eram fisicamente ativas, porém, considerando somente a AF praticada durante o lazer e avaliada no segundo trimestre gestacional. De qualquer maneira, comparações diretas se tornam limitadas devido às diferenças metodológicas na aferição dos dados sobre AF entre os estudos.

Alguns estudos também corroboram nosso achado de que ocorre diminuição gradativa da prática de AF conforme o avanço da gestação. Watson et al.^18^ mostraram que houve redução no nível de AF do segundo para o terceiro trimestre gestacional (12,8% para 9,7%). Ko et al.^19^ também mostraram que a média de AF autorreferida por meio de escala visual analógica foi maior no segundo trimestre (5,01 ± 1,99), com redução no terceiro (4,62 ± 1,97). Acredita-se que nos primeiros trimestres as gestantes se sentem mais confiantes para realizar AF, uma vez que se preocupam mais com a saúde do bebê e com seu ganho de peso. Já ao final da gestação é comum surgirem desconfortos excessivos e algumas contraindicações para o exercício físico, o que pode justificar o abandono da prática regular devido ao aumento de peso^20^. A inatividade física pré-gestacional aliada ao ganho de peso excessivo nos últimos meses da gestação, bem como os modelos sociais em relação ao comportamento físico adquiridos durante a infância, estão entre os fatores que levam à diminuição da prática de AF no decorrer do período gestacional^21^.

No presente estudo as puérperas não praticantes de AF durante o terceiro trimestre gestacional tiveram maiores chances de parto cesáreo. Um estudo conduzido em Taiwan verificou que a prática de AF no terceiro trimestre diferiu significativamente entre as mulheres que foram submetidas ao parto cesáreo não planejado e aquelas que tiveram parto normal, com maiores chances de cesarianas para as mulheres que apresentaram baixos níveis de AF no terceiro trimestre^19^. Em outro estudo com população de mulheres nulíparas residentes na Dinamarca foi encontrado que o nível crescente de AF no tempo de lazer se associou a um modo de parto menos complicado, ou seja, a menores probabilidades de realizar parto cesáreo de emergência ou extração à vácuo. Os autores sugerem que a AF no lazer até o final da gestação pode ter papel importante na redução do número de cesáreas de emergência e partos vaginais assistidos, o que é de grande interesse para a saúde pública, uma vez que a AF é um comportamento saudável, acessível e que causa pouco ou nenhum efeito negativo nas gestantes^22^. Rajabi et al.^23^ também mostraram menores probabilidades (OR = 0,68; IC95% 0,47–0,97) de realização de parto cesáreo em mulheres que aumentaram o nível de AF durante a gestação em comparação com o período pré-gravídico.

Mesmo sem considerar a avaliação de AF por trimestres gestacionais, uma metanálise realizada com 12 estudos concluiu que o exercício físico regular em níveis baixos ou moderados durante a gestação parece aumentar modestamente a chance de parto normal em gestantes saudáveis^3^. No presente estudo a prática de AF durante qualquer período da gestação, quando ajustada apenas para as características sociodemográficas e comportamentais e sem considerar as condições de risco para a saúde materna, também reduziu as chances de parto cesáreo. Corroborando com esses achados, Chia-Hui Chen et al.^24^ mostraram que a AF total, sem relação com os trimestres, aumentou as chances de parto vaginal (AOR = 1,017; p < 0,05). Além disso, tem sido observado que a prática de um programa estruturado de AF por gestantes pode diminuir o risco de cesarianas em 15%, evidenciando a importância de que as gestantes sejam encorajadas a se exercitarem de acordo com as recomendações do American College of Obstetricians and Gynecologists (ACOG)^25^.

No presente estudo, não praticar AF durante o terceiro trimestre de gestação também se associou significativamente às maiores chances de baixo peso ao nascer. No entanto, até o presente momento, há na literatura poucos estudos que testaram essa associação especificamente no terceiro trimestre gestacional. Nos achados de Clapp et al.^26^ as gestantes que praticavam AF a partir da 8ª semana de gestação, durante 20 minutos e de 3 a 5 vezes por semana, deram à luz a recém-nascidos mais pesados quando comparadas às gestantes sedentárias (3.750g–3.490g). No estudo de Regô et al.^8^ foi avaliado somente a AF praticada no segundo trimestre gestacional, e, mesmo assim, não se observou associação em relação ao baixo peso, independentemente do nível da AF das gestantes. Outros estudos populacionais realizados com grandes amostras de mulheres norueguesas^27^ e dinamarquesas^28^ também não identificaram associação entre a prática de AF na gestação e o baixo peso ao nascer. Por outro lado, observaram que a prática de AF de 3 a 5 vezes por semana durante o segundo e terceiro trimestres se mostrou protetora (OR = 0,82; IC95% 0,73–0,91) em relação à prematuridade^27^. E, embora este estudo não tenha evidenciado associações significativas entre a prática de AF (independente do trimestre gestacional) e a menor ocorrência de prematuridade, vale ressaltar que são desfechos comumente relacionados, já que os bebês que nascem prematuros também tendem a apresentar baixo peso.

No estudo de Haakstad et al.^21^ foram avaliadas gestantes no 2° e 3° trimestres de gravidez que praticaram exercício aeróbico duas vezes por semana durante 60 minutos por 12 semanas. Os achados não mostraram diferenças estatisticamente significativas para o peso médio e baixo peso ao nascer (< 2.500g), assim como a AF regular durante a gestação não ocasionou a prematuridade. Já em estudo mais recente, Beetham et al.^29^ observaram por meio de uma metanálise que as mulheres praticantes de AF vigorosa em qualquer período gestacional tiveram proteção para o baixo peso ao nascer através do risco relativo (RR = 0,44) e para a prematuridade (RR = 0,20). Vale considerar, no entanto, que as explicações fisiológicas da prática de AF na gestação e a ocorrência de partos prematuros ainda se mostram controversas. Por um lado, acredita-se que o estímulo ao nascimento prematuro pode ocorrer devido à maior liberação de catecolaminas e noradrenalina decorrentes do exercício físico, as quais induzem a atividade miometrial e aceleram o parto^10^. E, por outro, o fato de a AF ocasionar diminuição do estresse oxidativo e melhorar a vascularização da placenta poderia diminuir o risco de partos prematuros, além de estar associado a redução da incidência de diabetes mellitus gestacional e distúrbios hipertensivos^10^.

Diante desses achados, considera-se que, apesar da escassez de estudos corroborando os efeitos positivos da prática de AF de cada trimestre para os desfechos favoráveis do puerpério, torna-se bastante evidente a importância do engajamento em AF, especialmente ao final da gestação. O exercício físico regular é recomendado para gestantes saudáveis, tanto para as atletas (considerando adaptações nos treinos) quanto para aquelas que não foram fisicamente ativas no período pré-gestacional, as quais devem seguir um programa de exercícios com aumento gradativo da intensidade durante a gestação. Além de todos os benefícios relacionados ao recém-nascido, a prática de AF regular na gestação pode promover o bem-estar geral da mulher, ajudar na manutenção do ganho de peso adequado, reduzir distúrbios hipertensivos, risco de pré-eclâmpsia, trombose venosa profunda e diabetes gestacional^30^.

Considera-se também que, para melhor compreensão da relação entre a prática de AF nos períodos gestacionais e os desfechos no puerpério, é importante conhecer outros fatores como intensidade, duração, frequência e modalidade de exercício físico. Esses aspectos podem ser apontados como limitação do presente estudo, já que as puérperas avaliadas não foram questionadas a respeito de todas essas características. E, apesar dos nossos achados indicarem a importância da prática de AF gestacional (especialmente no terceiro trimestre) para o alcance de melhores desfechos no parto, vale ressaltar que algumas mulheres podem ter apresentado contraindicações absolutas para sua realização, as quais talvez não tenham sido identificadas nas nossas análises. Além disso, as informações foram subjetivas, o que pode ter subestimado ou superestimado o nível de AF relatado em decorrência da percepção e memória. Por fim, entende-se que caso as análises não tivessem sido realizadas conforme as macrorregiões do estado, poderiam refletir as disparidades culturais, geográficas, de implementação de políticas públicas, dentre outras, e viabilizar resultados mais específicos para as ações dos gestores de saúde locais.

Entretanto, deve-se considerar como ponto positivo do estudo o fato de ele ter sido realizado com amostra probabilística de puérperas de todo o estado de Santa Catarina e que realizaram o parto e o pré-natal exclusivamente pelo Sistema Único de Saúde, as quais costumam ser analisadas conjuntamente com as amostras das maternidades da rede privada. A avaliação da AF conforme o trimestre gestacional também merece destaque, uma vez que os achados fortalecem e apoiam a AF precoce na gestação, ao mesmo tempo que denotam a importância da sua realização até o momento do parto. Outro ponto relevante envolve o fato de o presente estudo utilizar dados empíricos sobre AF na gestação, um dos principais comportamentos de saúde que é passível de modificações, podendo contribuir para subsidiar ações e políticas públicas para melhoria da assistência pré-natal.

Mesmo com a relevância dos nossos achados, vale destacar que pouco mais de um quinto da amostra (20,6%) relatou praticar AF durante qualquer período gestacional, indicando que as mulheres que se mantêm ativas durante a gestação ainda representam uma minoria dentre todas que realizam o pré-natal na rede pública de Santa Catarina. Dessa forma, denota-se a relevância da temática para incentivar a prática de AF durante a gestação, a qual muitas vezes é negligenciada pelos profissionais e serviços de saúde. Também se considera importante fomentar novos estudos com esse público que visem identificar os principais fatores relacionados à oferta, acesso e atitudes que propiciam ou dificultam a adoção de comportamentos ativos e saudáveis durante a gestação.

Assim, concluiu-se que não praticar AF no terceiro trimestre da gestação aumenta as chances de parto cesáreo e de baixo peso ao nascer. Tais resultados sugerem a importância do incentivo à prática de AF por meio do fortalecimento das políticas públicas envolvendo as gestantes através de informação e acolhimento no sistema, proporcionando redução de gastos públicos hospitalares e proporcionando melhor qualidade de vida para a gestante e o bebê.
